# Development of 22 Polymorphic Microsatellite Loci for the Critically Endangered Morato’s Digger Toad, *Proceratophrys moratoi*

**DOI:** 10.3390/ijms131012259

**Published:** 2012-09-25

**Authors:** Maurício Papa Arruda, William Pinheiro Costa, Carla Cristina Silva, Shirlei Maria Recco Pimentel

**Affiliations:** 1 Department of Structural and Functional Biology, Institute of Biology, University of Campinas-UNICAMP, Campinas, SP, CEP 13083-863, Brazil; E-Mail: shirlei@unicamp.br; 2 Department of Zoology, Institute of Biosciences, State University of São Paulo-UNESP, Botucatu, SP, CEP 18618-970, Brazil; E-Mail: pinho_willi@hotmail.com; 3 Center for Molecular Biology and Genetic Engineering, Institute of Biology, University of Campinas-NICAMP, Campinas, SP, CEP 13083-875, Brazil; E-Mail: silvacbio@yahoo.com.br

**Keywords:** *Proceratophrys moratoi*, endangered species, microsatellite, population genetics, Morato’s digger toad, conservation genetics

## Abstract

The Morato’s digger toad (*Proceratophrys moratoi*) inhabits Brazilian moist savannas and is critically endangered due to its very limited geographic distribution, reduced number of isolated populations, and evidence of population decline and local extinctions. With the objective of providing tools for the genetic study of the species, 22 polymorphic microsatellite loci were isolated and screened using DNA extracted from samples of oral mucosa cells obtained from 113 individuals representing five remnant *P. moratoi* populations in the Brazilian state of São Paulo. These markers presented 2–18 alleles per locus, polymorphism information content (PIC) of 0.02–0.87, observed heterozygosity of 0.02–0.96 and expected heterozygosity of 0.02–0.87. Three of the loci deviated significantly from Hardy–Weinberg equilibrium in one of the populations, possibly due to the presence of null alleles. Significant linkage disequilibrium was also detected between three pairs of loci. The molecular markers developed in this study were able to discriminate each of the individuals sampled (identity analysis). This means that they will be extremely useful for future genetic studies applied to the conservation of *P. moratoi*, providing a baseline for estimating the levels of genetic diversity, pedigrees, inbreeding, and population structure, which will be essential for the development of effective genetic management programs.

## 1. Introduction

The class Amphibia has experienced a major global decline in recent decades, becoming more endangered than birds and mammals, due to a combination of factors [1]. Habitat destruction, climate change and infectious diseases are considered to be the primary cause of the decline of this group [2,3]. Ongoing anthropogenic impacts have contributed to the increasing deterioration of landscapes, which not only modifies aquatic and terrestrial habitats, but also reduces their connectivity, which are all factors that may affect amphibian populations adversely [4,5].

*Proceratophrys moratoi* is a digger toad of small size, typically with a snout-vent length of no more than 35 mm, which is endemic to the Cerrado savanna of the Brazilian state of São Paulo [6]. The species is found in *campo sujo* habitats (grassland dotted with small shrubs), invariably near the gallery forests associated with the headwaters of streams [6,7]. In São Paulo, the Cerrado biome has been modified intensively in recent decades, primarily for the planting of commercial crops such as sugarcane, but also for cattle ranching and urban development [8]. Currently, only about 6% of the original cover remains [9], which has drastically reduced the availability of potential habitat for the endemic *P. moratoi*. Due to its very restricted geographic distribution and the evidence of population decline and local extinctions [7,10], the species is currently listed as critically endangered by the International Union for Conservation of Nature [11], and is included in the official lists of endangered species of Brazil [12] and São Paulo [13].

The available genetic studies of *P. moratoi* include molecular analyses of mitochondrial and nuclear genes [14] and cytogenetics [15]. However, no population-level data—which may be essential for the development of effective management strategies—are available, due to the lack of appropriate molecular markers. In order to contribute to the development of these strategies, we have developed the first set of microsatellite markers for *P. moratoi*.

## 2. Results and Discussion

### 2.1. Characterization of the Enriched Microsatellite Library

A total of 384 clones were isolated and sequenced bidirectionally. The Codoncode Aligner 3.7.1 software (CodonCode Corporation: Centerville, MA, USA) revealed a redundancy of 17% in the library. Of the unique clones selected for analysis in Microsatellite Repeats Finder [16], 176 (46%) had at least one microsatellite. A predominance of dinucleotide repeats (56%) was found in the motifs that make up the library. The CA*_N_*/GT*_N_* repeats (130 motifs identified) were the most numerous, followed by CT*_N_*/GA*_N_* (48 motifs). This predominance of CA_N_/GT_N_ repeats is typical of the eukaryote genome [17]. Considerable numbers of other types of motifs were also recorded, in particular the dinucleotide AT*_N_*/TA*_N_* (36 motifs), the trinucleotides CAT*_N_*/GTA*_N_* (18 motifs), CTT*_N_*/GAA*_N_* (17), AAT*_N_*/TTA*_N_* and CTC*_N_*/GAG*_N_* (6 motifs each), and the tetranucleotides CTAT*_N_*/GATA*_N_* (13 motifs) and CATT*_N_*/GTAA*_N_* (6 motifs). These data provide a baseline that will support the development of additional probes for the isolation of new microsatellites in *P. moratoi*.

### 2.2. Development of Polymorphic Microsatellite Markers

A total of 29 pairs of primers were designed and optimized successfully for the PCR amplification of microsatellite loci (Table 1). Of the loci analyzed, *Pmoratoi*μ1 presented several nonspecific amplifications even after optimization (with varying concentrations of magnesium chloride and different annealing temperatures) and was excluded. Six loci—*Pmoratoi*μ2, *Pmoratoi*μ3, *Pmoratoi*μ4, *Pmoratoi*μ9, *Pmoratoi*μ20, and *Pmoratoi*μ22—were monomorphic. With the exception of *Pmoratoi*μ22, all these monomorphic loci represent interrupted or interrupted compound microsatellites characterized by a small number of repetitions, with predictably low polymorphism [18]. Twenty-two microsatellites were polymorphic (Table 2) in at least some populations (*Pmoratoi*μ7, *Pmoratoi*μ8, *Pmoratoi*μ10, *Pmoratoi*μ11, *Pmoratoi*μ14, *Pmoratoi*μ17, *Pmoratoi*μ18, and *Pmoratoi*μ21). The *Pmoratoi*μ5 locus was not amplified in some populations, possibly due to local mutations in the primer annealing site. The identity analysis calculated using Cervus 3.0.3 [19] detected four pairs of specimens with identical genotypes (exact match), suggesting the recapture of the same animal during fieldwork. In these cases, the duplicate genotype was excluded from the analyses.

The total number of alleles per locus (*N*_A_) varied between 2 and 18 (Table 2). Observed heterozygosity (*H*_O_) ranged from 0.02 to 0.96, expected heterozygosity (*H*_E_) from 0.02 to 0.87, and polymorphism information content (PIC) from 0.02 to 0.87. As might be expected from the relatively large number of markers developed for this study, evidence of linkage disequilibrium was found in three pairs of loci (*Pmoratoi*μ10-*Pmoratoi*μ25, *Pmoratoi*μ12-*Pmoratoi*μ15, and *Pmoratoi*μ15-*Pmoratoi*μ25) following Bonferroni correction (*p* < 0.002). Significant deviations from Hardy–Weinberg Equilibrium (HWE) were found in *Pmoratoi*μ24 and *Pmoratoi*μ27 from the São Carlos population and in *Pmoratoi*μ29 from Brotas, due to a deficit of heterozygotes. These deviations can be attributed to the presence of null alleles in these populations. The estimated null allele frequency for the *Pmoratoi*μ24 locus from São Carlos was 0.23, and that for *Pmoratoi*μ27 from this same population was 0.18. The estimated frequency for *Pmoratoi*μ29 from Brotas was 0.11.

In addition to the loci with deviations from HWE, null alleles were detected in the *Pmoratoi*μ13 (0.10), *Pmoratoi*μ23 (0.19), and *Pmoratoi*μ24 (0.10) loci from Bauru, and in *Pmoratoi*μ27 from Brotas (0.07). In all these cases, the evidence of the presence of null alleles was relatively weak and thus insufficient to confirm a significant departure from HWE following the Bonferroni correction. Micro-Checker 2.2.3 [20] did not detect small allele dominance, but found evidence of the presence of stutter bands in one locus, *Pmoratoi*μ13 from Bauru. The identity analysis indicated that the combination of all the loci would permit the individual identification of each of the specimens.

## 3. Experimental Section

### 3.1. Construction of Enriched Microsatellite Genomic Library

We constructed an enriched partial microsatellite genomic library using an approach based on the selective hybridization method of Kijas [21]. The library was constructed using DNA extracted from the muscle tissue of one specimen of *P. moratoi* using the procedure of Sambrook *et al*. [22] with modifications. Six micrograms of genomic DNA were digested with 50 units of *Afa* I (Invitrogen) and the fragments were then ligated to *Rsa* I linkers (*Rsa*21: 5′-CTCTTGCTTACGCGTGGACTA-3′/*Rsa*25: 5′-TAGTCCACGCGTAAGCAAGAGCACA-3′) using 2 units of T4 DNA ligase (Promega). The fragments were then amplified by polymerase chain reaction (PCR) with a reduced number of cycles (20 cycles) using the primer *Rsa*21. The PCR products were purified, denatured and hybridized with biotinylated microsatellite probes (GT_8_ and CT_8_) at room temperature for 20 min. The hybrid mixtures containing microsatellites were then collected by streptavidin-coated magnetic beads (Promega). The selected fragments were amplified via PCR and the products were ligated into a pGEM-T easy cloning vector (Promega). *Escherichia coli* XL1-Blue cells (Stratagene) were transformed with recombinant plasmids by electroporation and grown overnight in solid Luria-Bertani agar medium containing ampicillin, IPTG and X-Gal. The positive colonies were selected and grown in liquid medium with 2YT HMFM containing ampicillin. After growing for 16 h, they were stored at −80 °C.

### 3.2. Sequencing and Primer Design

Of the total of 596 clones obtained, 384 were sequenced bidirectionally in an ABI Prism 3100 automatic sequencer (Applied Biosystems: Foster City, CA, USA). The DNA sequences were exported into Codoncode Aligner 3.7.1 (CodonCode Corporation) which assembled the contigs and verified the redundancy of the library. The Bioedit program was used to check the quality of the sequences by chromatogram and to align them to form a consensus sequence. The repetitive elements were located using the Microsatellite Repeats Finder program [16]. After removal of the vector sequences, adapters, and restriction endonuclease sites by the Microsat software (version 1.0; CIRAD: Montpellier, France, 2005), the primers were designed using Primer 3 [23].

### 3.3. Genotyping

The polymorphic microsatellite markers were characterized by the amplification of the genomic DNA obtained from buccal epithelial cells (non-destructive method) following a modified version of the procedure described by Pidancier *et al*. [24]. Samples were obtained from five remnant *P. moratoi* populations in the Brazilian state of São Paulo: 41 samples were collected in the municipality of São Carlos (22°01′00.5″ S, 47°56′21.0″ W), 41 in Brotas (22°12′53″ S, 47°54′41″ W), 27 in Bauru (22°20′48.46″ S, 49°0′56″ W), 3 in Avaré (22°53.227′ S, 48°56.803′ W), and 1 in Lençóis Paulista (22°49′13.17″ S, 48°53′0.28″ W). The PCRs were prepared in a final volume of 15 μL containing 10 ng of the DNA template, 1× reaction buffer, 0.3 mM dNTP, 0.6–4.0 mM MgCl_2_ (Table 1), 0.6 μM of each primer, and 1 unit of Taq polymerase (Invitrogen). The reactions were conducted following the same cycling conditions: 5 min at 94 °C followed by 41 cycles of 30 s at 94 °C, 1 min at the locus-specific annealing temperature (Table 1), and 1 min at 72 °C, followed by a final extension of 30 min at 72 °C to minimize stutter bands. The PCR products were analyzed in a Dual Dedicated Height Sequencing Kit (CBS Scientific) vertical electrophoresis system in 6% denaturing polyacrylamide gel and stained with silver nitrate [25]. Allele size was estimated by comparison with a 10 bp DNA ladder (Invitrogen) and using the GelAnalyzer 2010a software [26].

### 3.4. Characterization of Polymorphic Markers

The levels of polymorphism of the microsatellites were evaluated as the number of alleles per locus (*N*_A_), observed (*H*_O_) and expected (*H*_E_) heterozygosity calculated by Popgene 1.32 [27]. The polymorphism information content (PIC) was calculated with Cervus 3.0.3 [19], which was also used to conduct a test of individual discrimination (identity analysis). The Genepop 4.0.9 software [28] was used to detect an excess or deficiency of heterozygotes, linkage disequilibrium between pairs of loci, and deviations from the Hardy–Weinberg Equilibrium (HWE), for which significance levels were determined using the Markov chain algorithm [29], with 10,000 dememorization steps, 100 batches and 5000 iterations per batch. All significance levels were adjusted by the sequential Bonferroni correction for multiple tests [30]. Micro-Checker 2.2.3 software [20] was used to identify genotyping errors and verify the presence of null alleles using the Brookfield method [31].

## 4. Conclusions

These are the first microsatellite markers developed for Morato’s digger toad, and in fact, the first for any member of the genus *Proceratophrys*. These markers constitute a powerful tool for the study of *P. moratoi* populations, allowing the identification of untagged individuals and providing a database for the development of kinship studies for future *ex situ* conservation programs. It will also be possible to analyze inbreeding, genetic diversity and structure, and gene flow in natural populations, which will be vital for the development of effective *in situ* conservation measures.

## Figures and Tables

**Table 1:** Microsatellites isolated in the present study with their respective primers and the optimal amplification conditions determined following visualization of the polymerase chain reaction (PCR) products in a polyacrylamide gel.

Genbank Accession n°	Locus	Repeat Motif	Primer Sequence (5′→3′)	T_A_	MgCl_2_ (mM)
JX441952	*Pmoratoi*μ1	(TTTC)_9_	Forward: GGTGAACATCCTTTTCGTAGC	50 °C	0.6
			Reverse: CACTCCTTCCCTAATCCAGTTT		
JX441953	*Pmoratoi*μ2	(AC)_4_AT(AC)_7_ (AC)_4_	Forward: ACACATCGTTCTGCACTACACAC	63 °C	1.0
			Reverse: GCTCCCTTGTCTTGCTGTCT		
JX441954	*Pmoratoi*μ3	(TA)_8_CACA CAT(AC)_8_	Forward: CTAACCGTCCAATAGCCTGTGT	63 °C	0.6
			Reverse: CCTCTTTCCCCTTGTGTGTCT		
JX441955	*Pmoratoi*μ4	(AC)_6_G(CA)_5_	Forward: AAATGAGGTGGCTGTGCTAAAT	60 °C	3.5
			Reverse: ATGCATTAGTGGTCATCACTGG		
JX441956	*Pmoratoi*μ5	(CA)_8_	Forward: TATCTGTATTGCCTGCTCCACAC	68 °C	3.5
			Reverse: CCTAGTGAGCTAAAAGTTGTGCTTGT		
JX441957	*Pmoratoi*μ6	(ACAT)_4_ (AC)_15_	Forward: CTGCACCACCCCTTGAATAA	46 °C	0.8
			Reverse: TGCACAGCAGGATCAATCTAAC		
JX441958	*Pmoratoi*μ7	(AC)_8_	Forward: ACTTCCAGGTGCCATATCTTCA	51 °C	1.0
			Reverse: AATTCTTGGTCTGCCATACTGTG		
JX441959	*Pmoratoi*μ8	(AC)_6_	Forward: GCGAATAATTGGAAAGCACAG	68 °C	3.5
			Reverse: GCCTGAGCCAGAGTTGAATAGTA		
JX441960	*Pmoratoi*μ9	(ATT)_4_…(TAT)_4_	Forward: GATAATTGACCGTTTCCGTCAT	63 °C	4.0
			Reverse: CATGGAACAAACTGAAGAGAACC		
JX441961	*Pmoratoi*μ10	(TA)_4_(CA)_7_	Forward: CTAATAAAGTGGCCGGTGAGTG	50 °C	0.8
			Reverse: ATAGGACTACATTGTGCCCTTGA		
JX441962	*Pmoratoi*μ11	(CA)_8_	Forward: TCCAAAGTTCTAGCCTTGTTAG	57 °C	4.0
			Reverse: CGCTACACATACCTTGAGAAA		
JX441963	*Pmoratoi*μ12	(ATCT)_7_ (CA)_5_…(CA)_4_	Forward: CCTTCCCACCTTCCCTCTC	66 °C	2.5
			Reverse: CGATCAACCTCCTCTTCTGTCTAC		
JX441964	*Pmoratoi*μ13	(CA)_7_	Forward: CTGTTTGGACTGCGATTCTT	50 °C	1.0
			Reverse: GCATTTGTGTGTGAGAGTGAA		
JX441965	*Pmoratoi*μ14	(ACAT)_8_	Forward: GTCAAATGAGGCGGCTGTG	63 °C	1.5
			Reverse: GCCATTATTGCTTGTATTGCTTCAG		
JX441966	*Pmoratoi*μ15	(GATA)_12_ (CA)_8_	Forward: CTTTAGGGCAGTCCAAGATTA	50 °C	1.5
			Reverse: TGAAGGGGACACATTTTAAG		
JX441967	*Pmoratoi*μ16	(TCA)_8_	Forward: CTACACTAAAACGTCTCAATCAATG	66 °C	2.5
			Reverse: ATGATGAAGAACTGGAGGAAGA		
JX441968	*Pmoratoi*μ17	(CAC)_7_	Forward: CCCAAAGAGTGCCAAGAAAATA	60 °C	0.8
			Reverse: GGTAACAAACAACAAACCAGTATCAAC		
JX441969	*Pmoratoi*μ18	(CA)_7_	Forward: GTGTAATCCTGGGGTTCAGGTA	57 °C	1.0
			Reverse: TCCCACCTTGGTCAGATATTGT		
JX441970	*Pmoratoi*μ19	(CA)_8_	Forward: TATAGTCCAGGCAGCCCCTTTA	68 °C	1.0
			Reverse: GTCCGTGAGTGACGCAAAGT		
JX441971	*Pmoratoi*μ20	(CA)_5_AG(CA)_6_	Forward: GATTCCCAGCAGAACATCAC	63 °C	0.8
			Reverse: GGACTATGGAGCAATGAAAGAA		
JX441972	*Pmoratoi*μ21	(CA)_4_…(CA)_4_… (CA)_5_…(CA)_7_	Forward: GGGGCACAGTGTATATGTCAGT	66 °C	3.0
			Reverse: TTGAGCTGGTGAGGCAGTT		
JX441973	*Pmoratoi*μ22	(TTTC)_17_	Forward: AAAATTCCGCTCAGTCATTA	42 °C	4.0
			Reverse: ACTCCTTCCCTAATCCAGTTT		
JX441974	*Pmoratoi*μ23	(TA)_4_(CA)_11_	Forward: ACCTGGTCTAACCCTTTGGAAAT	70 °C	3.0
			Reverse: CAGCGTTACCAGACATTTTATGTTC		
JX441975	*Pmoratoi*μ24	(AT)_7_	Forward: GCTATTTGTCTACCTATCTATCTTTCAT	40 °C	4.0
			Reverse: CAATAAAACTCTGGACCTTGAAC		
JX441976	*Pmoratoi*μ25	(ACT)_11_	Forward: TCTAATGTCCACACTGCTACTACT	70 °C	4.0
			Reverse: GCTAATGGCCGAGTTATTG		
JX441977	*Pmoratoi*μ26	(CA)_7_	Forward: ATTTGGCTGTCTGACCTGTCTTA	63 °C	4.0
			Reverse: CCCATATTAGTTCGGATCACAAG		
JX441978	*Pmoratoi*μ27	(TCTA)_17_	Forward: CTCTATCTAACCCTTTCATA	57 °C	2.0
			Reverse: AAGATGGATAGATGTGAGA		
JX441979	*Pmoratoi*μ28	(CA)_8_	Forward: GAAATGAGAGGCGTGAGAGAT	51 °C	1.0
			Reverse: GCTGTCCGTCAATGGGTAT		
JX441980	*Pmoratoi*μ29	(CA)_16_	Forward: GAGGAAAAGTCAAGGAACTAAATGTC	46 °C	0.8
			Reverse: ACAGTCTTCTCAATCTGCATGTCT		

T_A_: annealing temperature; MgCl_2_: magnesium chloride.

**Table 2:** Descriptive analysis of the genetic diversity in 22 polymorphic microsatellite loci obtained from five populations of *P. moratoi*. Significant deviations (*p* < 0.002) from Hardy–Weinberg Equilibrium (HWE) following the Bonferroni correction are indicated by an asterisk (*). Heterozyogosity and HWE were not estimated for the populations with small sample size (Avaré and Lençóis Paulista).

Population Locus	São Carlos (*n* = 41)			Bauru (*n* = 27)			Brotas (*n* = 41)			Avaré (*n* = 1)	LP (*n* = 3)	Total		

	*N*_A_	*H*_O_	*H*_E_	*N*_A_	*H*_O_	*H*_E_	*N*_A_	*H*_O_	*H*_E_	*N*_A_	*N*_A_	S	*N*_A_	PIC
*Pmoratoi*μ5	5	0.29	0.35	-	-	-	3	0.06	0.06	-	-	201–239	6	0.47
*Pmoratoi*μ6	8	0.70	0.71	5	0.62	0.68	9	0.59	0.68	2	1	208–240	10	0.78
*Pmoratoi*μ7	1	0.00	0.00	1	0.00	0.00	2	0.46	0.48	1	1	185–187	2	0.28
*Pmoratoi*μ8	2	0.17	0.16	1	0.00	0.00	2	0.02	0.02	1	1	209–211	2	0.06
*Pmoratoi*μ10	3	0.30	0.31	1	0.00	0.00	3	0.12	0.12	1	1	109–113	3	0.15
*Pmoratoi*μ11	2	0.05	0.05	1	0.00	0.00	3	0.12	0.14	1	1	125–129	3	0.07
*Pmoratoi*μ12	6	0.80	0.77	8	0.52	0.64	13	0.85	0.87	3	2	144–196	14	0.82
*Pmoratoi*μ13	2	0.12	0.16	3	0.42	0.59	2	0.05	0.05	2	1	162–168	4	0.34
*Pmoratoi*μ14	7	0.75	0.76	1	0.00	0.00	5	0.68	0.71	1	2	190–218	8	0.71
*Pmoratoi*μ15	10	0.67	0.80	9	0.83	0.79	12	0.73	0.85	2	2	177–241	18	0.84
*Pmoratoi*μ16	3	0.62	0.55	3	0.75	0.64	2	0.10	0.10	2	1	146–158	4	0.48
*Pmoratoi*μ17	3	0.08	0.08	2	0.04	0.04	1	0.00	0.00	1	1	092–098	3	0.09
*Pmoratoi*μ18	1	0.00	0.00	1	0.00	0.00	1	0.00	0.00	2	1	163–165	2	0.02
*Pmoratoi*μ19	2	0.50	0.51	2	0.46	0.49	2	0.36	0.43	2	1	167–169	2	0.40
*Pmoratoi*μ21	1	0.00	0.00	2	0.40	0.47	2	0.17	0.16	1	1	242–246	3	0.25
*Pmoratoi*μ23	9	0.72	0.86	4	0.29	0.61	7	0.76	0.81	3	1	225–251	12	0.87
*Pmoratoi*μ24	3	**0.26**	0.66 *	2	0.30	0.47	2	0.10	0.09	1	2	148–174	8	0.72
*Pmoratoi*μ25	3	0.60	0.55	3	0.57	0.58	2	0.54	0.51	1	2	246–252	3	0.53
*Pmoratoi*μ26	2	0.13	0.12	2	0.42	0.38	2	0.41	0.46	1	1	119–133	4	0.38
*Pmoratoi*μ27	8	**0.45**	0.77 *	8	0.87	0.84	9	0.69	0.85	4	2	196–244	12	0.86
*Pmoratoi*μ28	2	0.45	0.49	3	0.58	0.58	3	0.61	0.62	1	1	195–203	3	0.48
*Pmoratoi*μ29	8	0.90	0.84	7	0.96	0.77	6	**0.47**	0.64 *	3	1	154–198	11	0.80

LP: Lençóis Paulista; 0.00: monomorphic locus; -: locus not amplified; *N*_A_: number of alleles; *H*_O_: observed heterozygosity; *H*_E_: expected heterozygosity; S: size range; PIC: polymorphic information content.

## References

[1] Stuart S.N., Chanson J.S., Cox N.A., Young B.E., Rodrigues A.S.L., Fischman D.L., Waller R.W. (2004). Status and trends of amphibian declines and extinctions worldwide. Science.

[2] Cushman S.A. (2006). Effects of habitat loss and fragmentation on amphibians: A review and prospectus. Biol. Conserv.

[3] Hof C., Araújo M.B., Jetz W., Rahbek C. (2011). Additive threats from pathogens, climate and land-use change for global amphibian diversity. Nature.

[4] Semlitsch R.D., Semlitsch R.D. (2003). Conservation of Pond-Breeding Amphibians. Amphibian Conservation.

[5] Denoël M., Ficetola G.F. (2008). Conservation of newt guilds in an agricultural landscape of Belgium: The importance of aquatic and terrestrial habitats. Aquat. Conserv. Mar. Freshw. Ecosyst.

[6] Jim J., Caramaschi U. (1980). Uma nova espécie de *Odontophrynus* da região de Botucatu, São Paulo, Brasil (Amphibia, Anura). Rev. Bras. Biol.

[7] Brasileiro C.A., Martins I.A., Jim J. (2008). Amphibia, Anura, Cycloramphidae, *Odontophrynus moratoi*: Distribution extension and advertisement call. Check List.

[8] Instituto Florestal de São Paulo (2005). Inventário Florestal da Vegetação Natural do Estado de São Paulo.

[9] Secretaria de Estado do Meio Ambiente (1997). Cerrado: Bases Para Conservação e uso Sustentável das Áreas de Cerrado do Estado de São Paulo.

[10] Rolim D.C. (2009). Bioecologia de *Odontophrynus moratoi* (Amphibia, Anura, Cycloramphidae). Master Dissertation.

[11] Cruz C.A.G., Caramaschi U Odontophrynus moratoi. IUCN 2010.

[12] Ministério do Meio Ambiente (2008). Livro Vermelho da Fauna Brasileira Ameaçada de Extinção.

[13] Secretaria do Meio Ambiente (2009). Fauna Ameaçada de Extinção no Estado de São Paulo: Vertebrados.

[14] Amaro R.C., Pavan D., Rodrigues M.T. (2009). On the generic identity of *Odontophrynus moratoi* Jim & Caramaschi, 1980 (Anura, Cycloramphidae). Zootaxa.

[15] Soma M., Jim J., Ruiz I.R.G., Batistic R.F. (2006). Determination of karyotypes and nuclear DNA content in frogs on the Family Leptodactyldiae. Biol. Gen. Exper.

[16] Bikandi J (2006). Microsatellite Repeats Finder.

[17] Tóth G., Gáspári Z., Jurka J. (2000). Microsatellites in different eukaryotic genomes: Survey and analysis. Genome Res.

[18] Brandstrom M., Ellegren H. (2008). Genome-Wide analysis of microsatellite polymorphism circumventing the ascertainment bias. Genome Res.

[19] Marshall T.C., Slate J., Kruuk L.E.B., Pemberton J.M. (1998). Statistical confidence for likelihood-based paternity inference in natural populations. Mol. Ecol.

[20] Oosterhout C.V., Hutchinson W.F., Wills D.P.M., Shipley P. (2004). Micro-Checker software for identifying and correcting genotyping errors in microsatellite data. Mol. Ecol. Notes.

[21] Kijas J.M.H., Fowler J.C.S., Garbett C.A., Thomas M.R. (1994). Enrichment of microsatellites from the citrus genome using biotinylated oligonucleotide sequences bound to streptavidin-coated magnetic particles. Biotechniques.

[22] Sambrook J., Fritsch E.F., Maniatis T (1989). Molecular Cloning—A Laboratory Manual.

[23] Rozen S., Skaletsky H.J., Krawetz S., Misener S. (2000). Primer 3 on the WWW for General Users and for Biologist Programmers. Bioinformatics Methods and Protocols: Methods in Molecular Biology.

[24] Pidancier N., Miguel C., Miaud C. (2003). Buccal swabs as a non-destructive tissue sampling method for DNA analysis in amphibians. Herpetol. J.

[25] Creste S., Tulmann Neto A., Figueira A. (2001). Detection of simple sequence repeat polymorphism in denaturing polyacrylamide sequencing gels by silver staining. Plant. Mol. Biol. Rep.

[26] Lazar I., Lazar I (2010). Gel Analyzer 2010a: Freeware 1D gel electrophoresis image analysis software.

[27] Yeh F.C., Yang R., Boylet T (1997). POPGENE Version 1.32: Software Microsoft Window-Based Freeware for Population Genetic Analysis.

[28] Rousset F. (2008). GENEPOP’007: A complete re-implementation of the GENEPOP software for Windows and Linux. Mol. Ecol. Resour.

[29] Guo S.W., Thompson E.A. (1992). Performing the exact test of Hardy–Weinberg proportion for multiple alleles. Biometrics.

[30] Rice W.R. (1989). Analyzing tables of statistical tests. Evolution.

[31] Brookfield J.F.Y. (1996). A simple new method for estimating null allele frequency from heterozygote deficiency. Mol. Ecol.

